# Production of Glucose 6-Phosphate From a Cellulosic Feedstock in a One Pot Multi-Enzyme Synthesis

**DOI:** 10.3389/fbioe.2021.678038

**Published:** 2021-06-02

**Authors:** Anne Usvalampi, He Li, Alexander D. Frey

**Affiliations:** Department of Bioproducts and Biosystems, School of Chemical Engineering, Aalto University, Espoo, Finland

**Keywords:** ATP regeneration, cellulose valorization, enzymatic phosphorylation, glucose 6-phosphate, *Saccharomyces cerevisiae* hexokinase, *Pseudomonas aeruginosa* polyphosphate kinase 2

## Abstract

Glucose 6-phosphate is the phosphorylated form of glucose and is used as a reagent in enzymatic assays. Current production occurs via a multi-step chemical synthesis. In this study we established a fully enzymatic route for the synthesis of glucose 6-phosphate from cellulose. As the enzymatic phosphorylation requires ATP as phosphoryl donor, the use of a cofactor regeneration system is required. We evaluated *Escherichia coli* glucokinase and *Saccharomyces cerevisiae* hexokinase (HK) for the phosphorylation reaction and *Pseudomonas aeruginosa* polyphosphate kinase 2 (PPK2) for ATP regeneration. All three enzymes were characterized in terms of temperature and pH optimum and the effects of substrates and products concentrations on enzymatic activities. After optimization of the conditions, we achieved a 85% conversion of glucose into glucose 6-phosphate using the HK/PPK2 activities within a 24 h reaction resulting in 12.56 g/l of glucose 6-phosphate. Finally, we demonstrated the glucose 6-phosphate formation from microcrystalline cellulose in a one-pot reaction comprising *Aspergillus niger* cellulase for glucose release and HK/PPK2 activities. We achieved a 77% conversion of released glucose into glucose 6-phosphate, however at the expense of a lower glucose 6-phosphate yield of 1.17 g/l. Overall, our study shows an alternative approach for synthesis of glucose 6-phosphate that can be used to valorize biomass derived cellulose.

## Introduction

Glucose 6-phosphate is the phosphorylated form of glucose and is an essential reagent in enzymatic assays, for example to determine glucose 6-phosphate dehydrogenase (G6PDH) activity in patients that suffer from G6PDH deficiency. Current production occurs via a multistep chemical synthesis.

Biological production of glucose 6-phosphate could be achieved via two approaches, fermentation or enzymatic biosynthesis. Glucose 6-phosphate is an essential metabolite in the central carbon metabolism at the crossroad of glycolysis and the pentose phosphate pathway (Wilson, 1995, 2003). Typically, metabolites in the central carbon metabolism are consumed within seconds and do not accumulate. Furthermore, cellular membranes are impermeable for the negatively charged glucose 6-phosphate and other sugar phosphates and no specific transporters for these compounds exist in cells precluding excretion. Therefore, processes for enzymatic production of sugar-phosphates, such as xylulose-5-phosphates, 2-deoxyribose 5-phosphate or fructose 1,6-bisphosphate, have been established (Honda et al., 2010; Kim and Zhang, 2016; Wang W. et al., 2017). These achievements were spurred by the rapid developments in the field of enzymatic biomanufacturing (Dudley et al., 2015; You and Percival Zhang, 2017).

The intracellular phosphorylation of monosaccharides is catalyzed by hexokinases and glucokinases. Hexokinases share a common ATP binding core surrounded by more variable sequences and can be divided into four types. Type I to III are called low K_M_ isozymes due to their high affinity for glucose (Wilson, 1995, 2003). Type IV has different kinetic properties and serves a different function (Postic, 2001). Except for type IV enzymes, vertebrate hexokinases distinguish from plants and fungal hexokinases by size and are approximately of the double molecular weight due to fusion of two ancestral hexokinase proteins, resulting in so-called 50- and 100-kDa hexokinases, respectively (Bork et al., 1993). While 100-kDa hexokinases are strongly inhibited by glucose 6-phosphate, this feature is not shared with the 50-kDa hexokinases such as yeast hexokinase (Cárdenas et al., 1998). While hexokinases have a more relaxed sugar specificity, glucokinases are more specific for glucose. In bacteria the role of glucokinase is to phosphorylate any free glucose molecule, generated for example by the hydrolysis of disaccharides. Microbial glucokinases can be divided into three groups based on their structures and co-factor specificities (Hansen et al., 2002; Hansen and Schönheit, 2003; Sakuraba et al., 2004; Punta et al., 2012). Group II glucokinases are ATP-dependent glucokinases that have been identified in bacteria.

To date, several different ATP regeneration systems have been used (Andexer and Richter, 2015). The most common enzymatic methods for regenerating ATP from ADP/AMP are based on using polyphosphate/polyphosphate kinase, acetyl phosphate/acetate kinase and phosphoenolpyruvate/pyruvate kinase (Pollak et al., 1977; Shih and Whitesides, 1977; Resnick and Zehnder, 2000; Shiba et al., 2000; Andexer and Richter, 2015). Two types of polyphosphate kinases exist, polyphosphate kinase 1 (PPK1) and polyphosphate kinase 2 (PPK2). While PPK1 catalyzes the reversible transfer of the terminal phosphate group of ATP to form long chain polyphosphates, PPK2 enzymes catalyze the transfer of a phosphoryl group to nucleoside mono- or diphosphates (Andexer and Richter, 2015). PPK2 enzymes are classified into three subfamilies based on their substrate specificities (Motomura et al., 2014). Class I PPK2 catalyzes the transfer of a phosphoryl group from polyphosphate to nucleoside diphosphate, class II PPK2 catalyzes the transfer of a phosphoryl group from polyphosphate to the nucleoside monophosphate and class III PPK2 catalyzes nucleoside triphosphate synthesis from nucleoside mono- and diphosphates (Ahn and Kornberg, 1990; Nocek et al., 2008; Motomura et al., 2014).

In this study we generated an enzymatic route for biosynthesis of glucose 6-phosphate using cellulose as a cheap and highly abundant glucose donor and Graham’s salt as phosphate donor. We optimized the reaction conditions and determined the effects of substrates and products on glucose phosphorylation by *E*. *coli* glucokinase (EGlk) and yeast hexokinase (HK) and on the ATP regeneration using *P*. *aeruginosa* class I PPK2. We successfully demonstrated glucose 6-phosphate formation in a one-pot reaction. Finally, we generated a system for the concommittant enzymatic release of glucose from cellulose and its conversion into glucose 6-phospate using HK and PPK2.

## Materials and Methods

### Construction of Expression Constructs

The *P. aeruginosa* gene PA2428 encoding PPK2 was codon-optimized for expression in *E*. *coli* and purchased from Thermo Fisher Scientific GeneArt. It was received in the expression plasmid pET100/D-TOPO and named pAU005. The gene is expressed under control of T7 promoter with an N-terminal His-tag, X-press epitope and EK-recognition site for tag cleavage. The *glk* gene encoding *E*. *coli* glucokinase (EGlk) was PCR amplified from genomic DNA of *E. coli* (strain K12) using the oligonucleotides EGlk-Forward (5′-AT ACCATGGGTACAAAGTATGCATTAGTCGG-3′) and EGlk-Reverse (5′-AGACCTTAGGTCACATTCTGGGTTCTGGACAT CATCACCATCATCACTAAGAATTCATA-3′). The forward primer introduced a *Nco*I site and an extra codon for glycine at the 5′-end of the coding sequence of EGlk. The reverse primer appended a Gly-Ser-Gly linker and a 6x His tag, stop codon and *Eco*RI restriction site near the 3′-end. The purified PCR fragment was cloned into *Nco*I and *Eco*RI sites of vector pTrcHis2B (Invitrogen) generating plasmid pAU007. EGlk is expressed under control of Trc promoter. The construct was verified by DNA sequencing. Plasmids were transformed into *E. coli* BL21 (DE3) strain and transformants were selected from LB agar plates containing 100 mg/L of ampicillin.

### Expression and Purification of Recombinant EGlk and PPK2

An overnight culture of *E. coli* BL21 (DE3) carrying pAU005 was diluted 1:50 into 1 L of LB supplemented with 100 mg/L ampicillin. Expression cultures were grown at 37°C, 220 rpm. When cultures reached an OD of 0.45, the cultures were induced with 1 mM IPTG and protein expression was conducted at 15°C for 22 h. The cells were collected by centrifugation (3,900 g, 10 min at 4°C) and stored at −20°C.

An overnight culture of *E. coli* BL21 (DE3) carrying pAU007 was diluted 1:50 into 500 ml of LB supplemented with 100 mg/L ampicillin. Expression cultures were grown at 37°C, 220 rpm. When cultures reached an OD of 0.6, the cultures were induced with 1 mM IPTG and protein expression was conducted at 15°C for 18 h. The cells were collected by centrifugation (3,900 g, 10 min at 4°C) and stored at −20°C.

The cell pellets were resuspended in 50 mM NaH_2_PO_4_, 50 mM NaCl, 10 mM imidazole, pH 7.4 containing protease inhibitor (Complete tablets, EDTA-free, Roche). Cells were disrupted by sonication for 4 min (cycles of 2 s sonication and 2 s pause). Cell debris were removed by centrifugation and the cleared lysate was filtered through 0.45 μm syringe filter (Whatman^®^ Puradisc).

The proteins were purified using Ni-NTA affinity chromatography on an Äkta Purifier system. A 1-ml HisTrap^TM^ Fast Flow Crude column (GE Healthcare) was equilibrated with 20 column volumes of 50 mM NaH_2_PO_4_, 50 mM NaCl, 10 mM imidazole, pH 7.4. The proteins were recovered by elution with a linear imidazole gradient from 10 to 500 mM over 20 min in 50 mM NaH_2_PO_4_, 50 mM NaCl, pH 7.4. Eluted fractions were tested for the presence of enzymatic activity and purity was checked on 12% SDS-PAGE gels. Fractions containing the protein of interest at sufficient purity were pooled and concentrated using Vivaspin 20 centrifugal concentrator (MWCO 10 kDa, Sartorious) The concentrated enzymes were stored in 50 mM sodium phosphate buffer, pH 7.5.

### Glucokinase and Hexokinase Activity Assay

The activity and stability of the purified EGlk and a commercial yeast hexokinase (HK) preparation (Hexokinase from *Saccharomyces cerevisiae* containing predominantly PII-isoform, Sigma-Aldrich H6380) were determined in a coupled enzyme assay adapted to a 96-well plate format in which the product of the kinase reaction, glucose 6-phosphate, is converted into 6-phospho-D-glucono-1,5-lactone with concomitant formation of NADH. The reaction was carried out in 50 mM sodium phosphate buffer (pH 7.5) containing 10 mM MgCl_2_, 3.75 mM NAD^+^, 1 mM ATP, 200 mM glucose and 1 U/ml G6PDH from *Leuconostoc mesenteroides* (Sigma) in presence of 10–20 mU/ml of enzyme. Temperature and pH optima, thermostability, and the kinetic parameters of EGlk and HK were determined in a 10 min reaction at 30°C. To determine the thermostability, EGlk and HK in 50 mM sodium phosphate buffer, pH 7.5 were incubated at temperatures ranging from 16 to 47°C for 24 h, after which the residual enzyme activity was measured. For determination of activating or inhibiting effects, glucose, ATP, ADP and polyphosphate were added to the reactions at the indicated concentrations. The apparent K_M_ and V_max_ values for glucose and ATP were determined in a reaction containing either 0–200 mM glucose and 10 mM ATP or 0–20 mM ATP and 200 mM glucose. The K_M_ and V_max_ were calculated using an online calculation tool (zunzun.com).

### PPK2 Activity Assay

The activity of PPK2 was determined by measuring the production of ATP with a luminescence assay in a 96-well plate format. PPK2 activity was determined in a reaction containing 10 mM sodium polyphosphate (Graham’s salt, Merck), 5 mM ADP in the reaction buffer (50 mM ammonium sulfate (NH_4_)_2_SO_4_, 50 mM Tris-HCl, 10 mM MgCl_2_, pH 7.5) and was started by addition of 0.01–0.3 U/ml PPK2. The reaction was carried out at 30°C for 10 min. Reaction mixtures were filtered to stop the reaction using centrifugal concentrators (Vivaspin 500; 10,000 MWCO) at 10,000 rpm for 5 min. The ATP concentration in the flowthrough was determined using Luciferase ATP determination assay kit (Invitrogen). The pH and temperature optima, thermostability, as well as the kinetic parameters of PPK2 were determined in sodium phosphate buffer, pH 7.5, 10 mM MgCl_2_, 10 mM sodium polyphosphate and 5 mM ADP in a 30-min reaction at 30°C. To determine the thermostability of PPK2, the enzyme in 50 mM sodium phosphate buffer, pH 7.5 was incubated at temperatures ranging from 16 to 47°C for 24 h, after which the residual activity was measured. For determination of activating or inhibiting effects, glucose, glucose 6-phosphate, ADP and polyphosphate were added to the reactions at the indicated concentrations. The apparent K_M_ and V_max_ values pf PPK2 for ADP were determined in a reaction containing 0–20 mM ADP. The K_M_ and V_max_ were calculated using an online calculation tool (zunzun.com).

### Differential Scanning Fluorometry

The structural stability of the enzymes was determined using differential scanning fluorometry (DSF). The optimal ratio of protein (0.2, 0.4, and 0.6 mg/ml) and SYPRO Orange dye (Thermo Fisher Scientific S-6650) (1x, 5x, 10x) for DSF measurement was optimized in a buffer containing 50 mM ammonium sulfate (NH_4_)_2_SO_4_, 50 mM Tris-HCl, 10 mM MgCl_2_, pH 7.5. After determination of optimal protein-to-dye ratio, the melting points of enzymes were determined in three different buffers (50 mM Tris-HCl, 50 mM HEPES, 50 mM sodium phosphate buffer) covering a pH-range between 6 and 9. The temperature cycle was conducted using a CFX96 connect Real-time PCR system (Bio-Rad). The method was designed to raise temperature from 20 to 90°C in 0.3°C increments with a 30-s hold for equilibration and fluorescence measurement in the FRET channel. The raw data were analyzed using the Bio-Rad CFX Manager software.

### High-Performance Anion-Exchange Chromatography With Pulsed Amperometric Detection

The concentration of glucose, cellobiose and glucose 6-phosphate were analyzed using a Dionex ICS-5000 ion chromatography system (Thermo Fisher Scientific, Waltham, United States) equipped with a CarboPac^TM^ PA200 IC column (3 × 250 mm) and a PA200-precolumn (3 × 50 mm). The column temperature was set to 40°C. The injection volume was 10 μl and a flow rate of 0.5 ml/min was applied. 100 mM NaOH (solution A) and 1 M NaOAc in 100 mM NaOH (solution B) were used as eluents. The starting condition was 100% solution A, followed by a 15-min gradient to 16% solution B, a 10-min gradient to 50% solution B and a 5-min gradient to 100% solution B. Between cycles the column was washed with 100% solution B for 5 min and equilibrated back to 100% solution A. 100 mg/L solution of glucose, cellobiose and glucose 6-phosphate were used to prepare standards. The data was processed with Chromeleon 7.2 software (Thermo Fisher Scientific).

### One Pot Production of Glucose 6-Phosphate From Glucose

Initial tests for optimizing HK and PPK2 enzyme ratio were conducted using the coupled enzyme assay. The reactions were carried out in 50 mM sodium phosphate buffer, pH 7.5, containing 10 mM MgCl_2_, 5 μM ATP, 25–100 mM glucose, 25–100 mM polyphosphate and supplemented with 3.75 mM NAD^+^ and 1 U/ml G6PDH. Varying amounts of HK (1.25–125 mU/ml) while keeping PPK2 activity constant (12.5 mU/ml) were used. Reaction mixtures were incubated at 30°C for 2 h.

The reaction for glucose 6-phosphate production included 0.3125 U/ml of HK and 0.115 U/ml of PPK2, respectively, in 50 mM sodium phosphate buffer, pH 7.5, 10 mM MgCl_2_ containing 50 mM glucose, 50 mM polyphosphate and 2 mM ATP. Experiments with feeding 50 mM glucose 6 h after start of the reactions were included. The reaction was carried out on a rolling platform reactor at 30°C for 24 h. Samples were heat inactivated at 100°C for 10 min and stored at −20°C until analysis. Enzymes were removed from samples prior to analysis using 1 ml C18 resin solid phase extraction columns (Supelclean^TM^ ENVI-18^TM^ SPE Tube, Sigma Aldrich & Merck).

### Cellulose Hydrolysis

Enzymatic glucose release from two cellulose model substrate was tested, the substrates were microcrystalline cellulose (Sigmacell, Type 101) and Whatman filter paper. For the enzymatic hydrolysis reaction *Aspergillus niger* sp. cellulase (Sigma) was selected. The amount of glucose and cellobiose released was tested over a period of 24 h at different substrate (2, 4, and 8%) and enzyme (1, 10, 50, 100 U/ml) concentrations. Glucose and cellobiose concentrations were determined as described above.

### One Pot Production of Glucose 6-Phosphate From Cellulose

Reaction mixtures for glucose 6-phosphate production from cellulose included 0.3125 U/ml of HK and 0.115 U/ml of PPK2, respectively, in 50 mM sodium phosphate buffer, pH 7.5, 10 mM MgCl_2_ containing 50 mM polyphosphate and 2 mM ATP, 2 or 4% of microcrystalline cellulose fibers and 10 or 50 U/ml *A. niger* sp. cellulase. The reaction and sample preparation were carried out as described above.

## Results

### Overexpression and Purification of the Recombinant Enzymes

Enzymatic phosphorylation of glucose using either hexokinases or glucokinases requires the cofactor ATP as phosphoryl donor and therefore the use of a cofactor regeneration system is required for production of glucose 6-phosphate at larger scale. We evaluated two different enzymes for the phosphorylation of glucose, a commercially available yeast hexokinase (HK) preparation and *E*. *coli* glucokinase (EGlk). The HK preparation contains predominantly the PII-isoform that is encoded by *HXK2* gene (Randez-Gil et al., 1998). Cloned and purified EGlK has been studied in detail (Meyer et al., 1997). The *E. coli glk* gene encodes a protein of 321 residues with a monomeric mass of 35 kDa that forms a dimer *in vivo*. Both selected enzymes are not inhibited by glucose 6-phosphate. For ATP recycling we chose the class I PPK2 encoded by *P. aeruginosa* PA2428 gene although the enzyme is less well characterized than the class I PPK2 encoded by PA0141. The selected PPK2 was previously used for GTP and ATP regeneration using linear polyphosphate (Andexer and Richter, 2015; Rexer et al., 2018).

**FIGURE 1:**
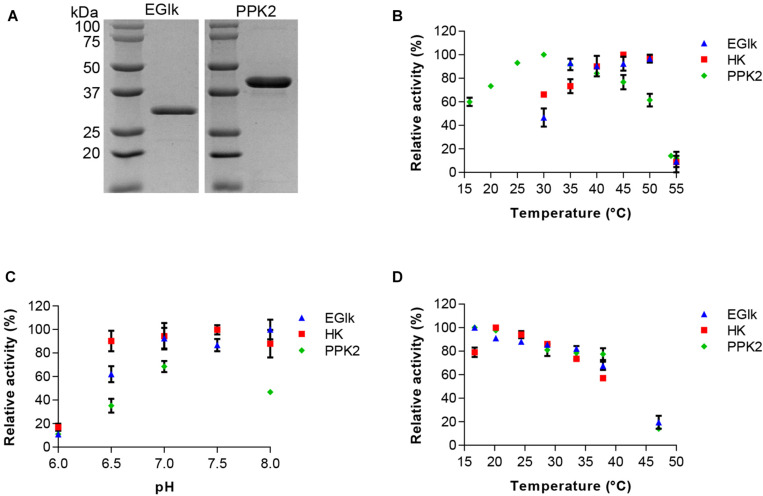
Characterization of HK, EGlk, and PPK2 enzymes. **(A)** Coomassie stained SDS-PAGE depicting recombinantly produced and purified EGlk and PPK2 enzymes. Characterization of HK, EGlk and PPK2 enzymes in terms of **(B)** temperature and **(C)** pH-optimum and **(D)** temperature stability. Residual enzyme activity was determined after a 24-h incubation at the indicated temperatures. EGlk and HK activities were determined using a coupled enzyme assay. PPK2 activity was determined using a Luciferase ATP determination assay. All data represent mean and standard error of the relative activities measured in triplicates. Where not visible, the error bar is smaller than the symbol size.

The EGlk and PPK2 enzymes were recombinantly produced in *E*. *coli* BL21 (DE3) and purified to homogeneity using affinity chromatography. Figure 1A) shows a 12% SDS-PAGE gel revealing single bands of EGlk and PPK2, respectively, with apparent molecular weights of approximately 35 and 39 kDa, respectively, corresponding to the expected molecular weights.

We used differential scanning fluorometry for an initial characterization of the stability of the enzymes in different buffer system that were considered most suitable for storage and the reactions. We observed a stabilizing effect of sodium phosphate buffer on the enzymes as the melting temperature (T_m_) of all enzymes increased compared to either Tris-HCl or HEPES buffered conditions. The T_m_ of EGlk, PPK2, and HK were 51.7 ± 1.03°C, 57.0 ± 0.4°C, and 41.0 ± 0.4°C in sodium phosphate buffer, pH7.5. HK was found to be the thermally most sensitive enzyme and the stabilizing effect of sodium phosphate buffer was only minor. Due to its stabilizing effect, phosphate buffer was chosen for storage of the enzymes and all further enzyme reactions.

### Characterization of the Enzymes and Determination of Kinetic Parameters

We determined the activity profiles of the three enzymes at pH ranging from 6 to 8 and temperatures ranging from 16 to 55°C. We observed that PPK2 exhibited a narrow pH tolerance reaching the highest activity at pH 7.5. In contrast HK was active over a wide pH range (pH 6.5–8.0). EGlk was active above a pH of 7, but rapidly lost activity at pH values lower than pH 7.0 (Figure 1B). The optimal reaction temperatures for the three enzymes varied, with an optimal temperature of 30°C for PPK2, 45°C for HK and 50°C for EGlk (Figure 1C). While PPK2 retained 76 and 61% of its maximal activity at 45 and 50°C, respectively, HK and EGlk activities reached 66 and 46% of their maximal activity at 30 °C, the optimal temperature of PPK2. Finally, as the glucose 6-phosphate production would run over a longer time, we also determined the residual enzyme activity after incubating the enzymes at temperatures ranging from 15 to 47°C for 24 h. The results indicated that all the enzymes were fairly stable as they retained 80% of their maximal activity up to a temperature of 35°C, after which the activity loss was stronger (Figure 1D). Based on these results, a reaction temperature of 30°C and a pH of 7.5, respectively, were selected.

Finally, we determined the apparent K_M_ and V_max_ under the conditions considered most suitable for the phosphorylation and ATP regeneration reactions (Table 1). The apparent V_max_ as a function of ATP and glucose concentrations were approximately 10 times higher for EGlk than for HK. The apparent K_M_ for ATP was lower and the apparent K_M_ for glucose was higher than the corresponding values for the HK. The apparent K_M_ and V_max_ of PPK2 were 0.023 ± 0.0059 mM and 0.00042 ± 0.00002 mM/min as a function of ADP concentrations.

**TABLE 1:** Kinetic parameters of yeast hexokinase and *E*. *coli* glucokinase.

	**Yeast hexokinase**		***E*. *coli* glucokinase**	
	**K_m_ (mM)**	**V_max_ (mM/min)**	**K_m_ (mM)**	**V_max_ (mM/min)**
Glucose	0.195 ± 0.012	0.0289 ± 0.000	1.494 ± 0.136	0.307 ± 0.0134
ATP	1.107 ± 0.074	0.0330 ± 0.008	0.566 ± 0.0313	0.282 ± 0.006

### Effect of Substrate and Product Concentrations on the Production of Glucose 6-Phosphate and ATP

**FIGURE 2:**
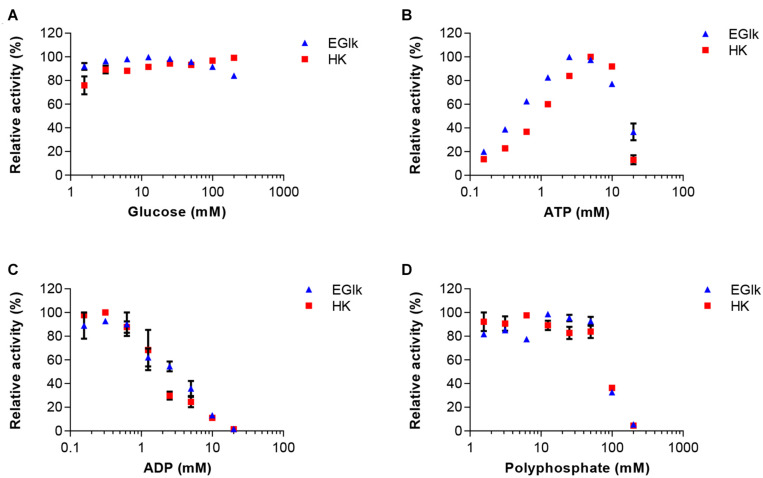
Effect of increasing concentrations of the reaction substrates **(A)** glucose and **(B)** ATP, the reaction product **(C)** ADP and **(D)** polyphosphate on EGlk and HK activities. EGlk and HK activities were determined using a coupled enzyme assay. All data represent mean and standard error of the relative activities measured in triplicates, except for determining the effect of ADP where the results were based on duplicate experiments. Where not visible, the error bar is smaller than the symbol size.

As in the final one-pot reaction multiple substrates and products will be present that could influence the reactions rates of other enzymes, we explored how these compounds supplied at different concentrations affected the reactions. In a first set we tested the effects of 0.15–20 mM ATP and ADP, and 1.5–200 mM glucose or polyphosphate on HK and EGlk activities. The relative reaction velocity of HK and EGlk was not affected by glucose (Figure 2). On the other hand, both enzymes showed inhibition when the polyphosphate concentration was raised, and the observed reaction rates rapidly dropped at concentrations over 50 mM. The reaction rate of both enzymes increased with increasing concentrations of ATP reaching a maximum between 3 and 5 mM ATP, but higher concentrations led to an inhibition of the reactions. ADP lowered the reaction even at small concentrations.

**FIGURE 3:**
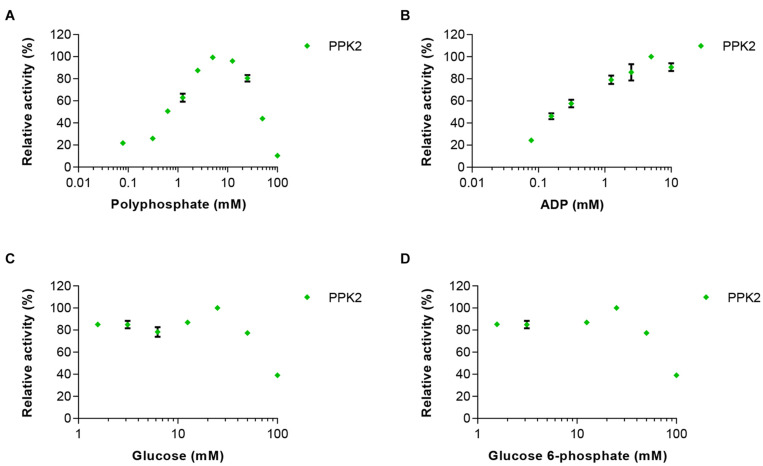
Effects of increasing concentrations of the reaction substrates **(A)** polyphosphate and **(B)** ADP on PPK2 activity. In addition, the effects of **(C)** glucose and **(D)** glucose 6-phosphate on enzyme activity were tested. PPK2 activity was determined using a Luciferase ATP determination assay. All data represent mean and standard error of the relative activities measured in triplicates. Where not visible, the error bar is smaller than the symbol size.

In the second set of experiments, the effects of 0–100 mM glucose, glucose 6-phosphate or polyphosphate on PPK2 activity was analyzed. In addition, ADP was used at concentrations between 0.15 and 10 mM. PPK2 activity was not strongly affected by increasing concentrations of glucose 6-phosphate, however, an inhibitory effect was observed when increasing glucose concentrations reaching only 40% activity at the highest glucose concentration tested (Figure 3). ADP had a clear activating effect on PPK2 activity at low concentration and the maximal activity was obtained at 5 mM. Polyphosphate activated PPK2 at low concentrations but resulted in activity loss at concentrations higher than 5 mM with the enzyme reaching only 50% of its maximal activity at 50 mM polyphosphate.

Based on these experiments we concluded that maintaining the correct concentration of ADP is very important, as too high concentrations would inhibit HK and EGlk, but low ADP concentrations led to reduced PPK2 activity and thus low ATP levels. Furthermore, polyphosphate and glucose 6-phosphate had significant effects on ATP production.

### One-Pot Glucose 6-Phosphate Production From Glucose

**FIGURE 4:**
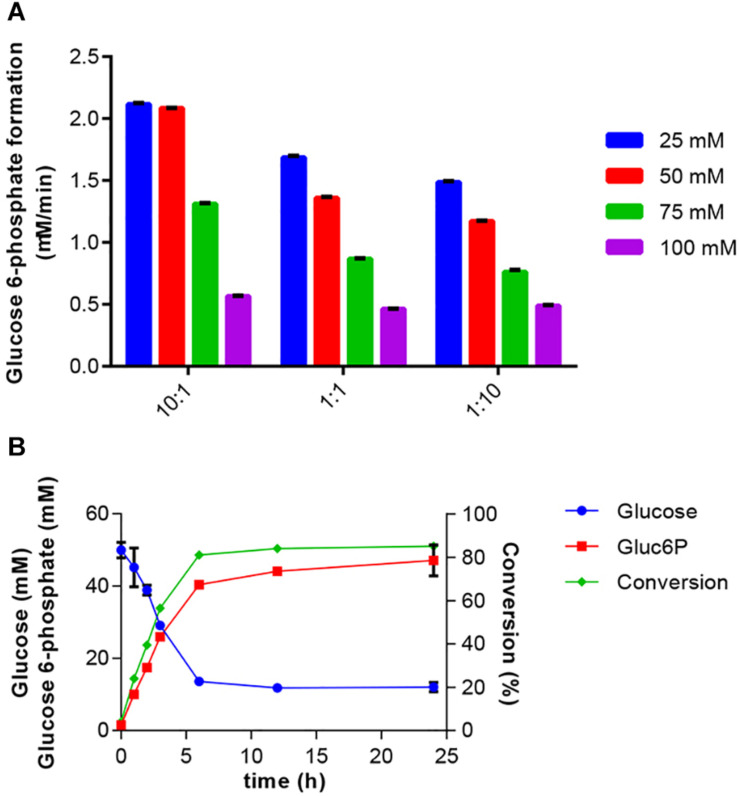
Production of glucose 6-phosphate. **(A)** Effect of HK to PPK2 ratio on glucose 6-phosphate production at different glucose and polyphosphate concentrations. Enzyme ratios used were 10:1, 1:1, and 1:10 and were calculated based on V_max_ values. The glucose 6-phosphate production was calculated after a 2 h reaction at 30°C. Glucose 6-phosphate production rate was determined using the coupled enzyme assay. **(B)** One-pot synthesis of glucose 6-phosphate production from glucose using HK and PPK2 activities. Reaction mixtures were incubated at 30°C and samples were taken at the indicated times. Produced glucose 6-phospate and remaining glucose were determined using HPAEC-PAD. Conversion indicates the fraction of glucose converted into glucose 6-phosphate. All data represent the mean and standard error of two or three replicates. If not visible, the error bar is smaller than the symbol size.

Initial tests were done with EGlk and HK. These experiments showed that EGlk was very unstable in diluted form as it rapidly lost activity. Therefore, HK was chosen for the two-enzyme reaction. Furthermore, HK activity was less sensitive to varying ATP concentrations and had a higher relative activity at 30°C, the optimal temperature for PPK2. Preliminary experiments were done in order to determine the ratio of HK to PPK2 and the initial concentrations of polyphosphate and glucose in the reactions. We tested the effects of different substrate concentrations ranging from 25 to 100 mM maintaining equimolar ratios of glucose and polyphosphate, while providing a fixed amount of ATP to the reaction. These tests were done at three different ratios of HK and PPK2 activities that were calculated based on the V_max_ of the two enzymes. Glucose 6-phosphate production rate was highest with a 10-fold excess of HK activity over PPK2 activity, while equal amounts of enzymes or PPK2 excess led to a lower production rate and were accompanied by a more rapid decrease in glucose 6-phosphate production. These results confirm our earlier experiments showing that especially higher polyphosphate concentrations inhibited both HK and PPK2 activities and glucose had an inhibitory effect on PPK2 (Figure 4A).

Bringing together the results of the previous experiments, the two-enzyme system for production of glucose 6-phosphate was set up. The reaction included 50 mM glucose, 50 mM polyphosphate and 2 mM ATP. The reaction was started by addition of 0.3125 U/ml HK and 0.115 U/ml PPK2 and samples for determination of glucose and glucose 6-phosphate concentrations were taken over a 24-h period (Figure 4B). A rapid decrease in substrate and increase in product concentrations were observed during the first 6 h after which only a small increase in product concentration was observed. Addition of glucose 6 h after the reaction start did not further increase glucose 6-phosphate production (data not shown). In the reaction a maximal conversion of glucose into glucose 6-phosphate of 85% was obtained.

### One-Pot Glucose 6-Phosphate Production From Cellulose

**FIGURE 5:**
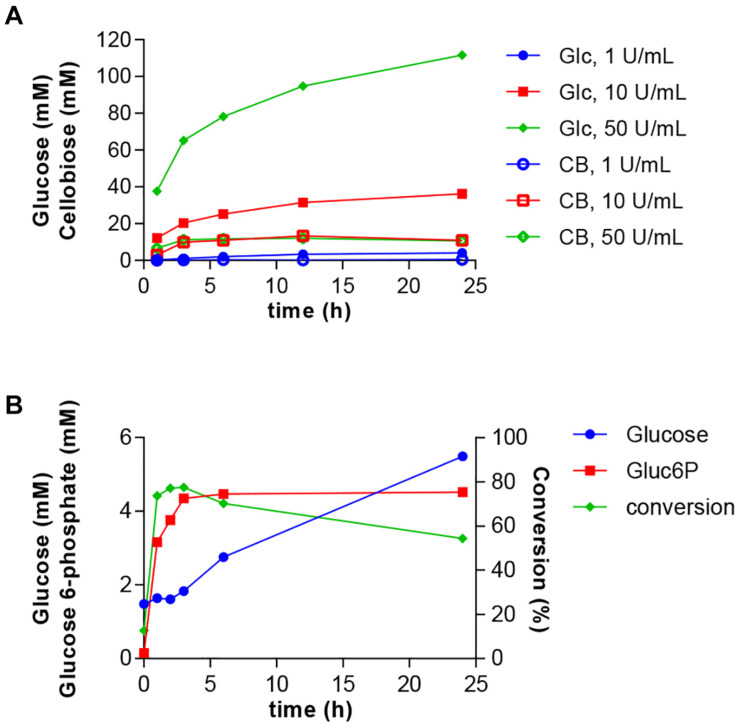
One-pot synthesis of glucose 6-phosphate from cellulose. **(A)** Optimization of glucose release from 2% microcrystalline cellulose by *A*. *niger* cellulase at different enzyme concentrations. Glucose (Glc) and cellobiose (CB) accumulation were determined over a 24-h period. **(B)** Cellulase, HK and PPK2 activities were combined in a reaction containing 2% cellulose, 2 mM ATP and 50 mM polyphosphate. Reactions were incubated at 30°C and samples were taken at the indicated times. Produced glucose 6-phospate and remaining glucose were determined using HPAEC-PAD. Conversion indicates the fraction of glucose converted into glucose 6-phosphate. All data represent the mean and standard error of two or three replicates. If not visible, the error bar is smaller than the symbol size.

Next, we intended to integrate the simultaneous glucose release from cellulose with the HK/PPK2 module. Such a process would enable the valorization of biomass residues into a value-added product. *A*. *niger* cellulase and most other cellulolytic enzymes are active at acidic pH. Therefore, we conducted experiments to determine the glucose release from cellulose by *A*. *niger* cellulase under the experimental conditions established for the glucose 6-phosphate production. The glucose release should be balanced with the glucose 6-phosphate production and not lead to excessive accumulation of glucose. Therefore, we tested different ratios of cellulose concentrations to cellulase activities. Based on these experiments we selected a cellulose concentration of 2% and tested glucose and cellobiose release using 1–50 U/ml of cellulase (Figure 5A). While the glucose release was highest at 50 U/ml, the use of 10 U/ml of cellulase led to a steadier glucose release over time. Under all tested conditions, only small amounts of cellobiose were produced.

The composition and conditions of the reaction were the same as in the two-enzyme reaction except for the use of 2% cellulose instead of 50 mM of glucose and the addition 10 U/ml of cellulase. Glucose was continuously produced through cellulose hydrolysis during the 24 h reaction (Figure 5B). The production of glucose 6-phosphate was fast during the first hours and reached a conversion rate of 77% after 6 h. After this time, despite availability of glucose, no more glucose 6-phosphate was produced. Compared to the two-enzyme system, the three-enzyme system showed the faster and higher conversion rate in the first hours, but the synthesis of glucose 6-phosphate rapidly ceased after 3 h of reaction. Therefore, we adjusted cellulose and cellulase concentrations. Although the altered conditions led to improved glucose release and a higher concentration of glucose 6-phosphate of up to 17.7 mM, the overall conversion rates dropped to less than 20% (data not shown).

## Discussion

Chemical synthesis of glucose 6-phosphate has a very long history (Seegmiller and Horecker, 1951) and currently, glucose 6-phosphate is commercially produced in synthetic reactions for example by using dibenzyl chlorophosphonate and monoacetone glucose in pyridine^1^. Alternatively, biological production of glucose 6-phosphate could be achieved through fermentation or enzymatic synthesis. Production of glucose 6-phosphate through fermentation is accompanied with several challenges, including its rapid turnover in cells due to its central role in the metabolism, the impermeability of membranes and the lack of specific transporters. One the other hand, the enzymatic manufacturing requires the supplementation of equimolar amounts of a phosphoryl-donor and glucose. To overcome these limitations, we developed a system for the enzymatic synthesis of glucose 6-phosphate using an ATP-dependent glucose-active kinase and a polyphosphate kinase-based ATP regeneration system.

We determined the kinetic parameters HK and EGlk at the experimental conditions considered optimal for the combined one-pot reaction that deviate from the optimal reaction conditions of each enzyme. The apparent K_m_ values of EGlk for glucose and ATP were 1.49 ± 0.14 and 0.57 ± 0.03 mM, respectively, and these values differ from earlier published K_m_ values of 0.78 mM for glucose and 3.76 mM for ATP (Meyer et al., 1997). The apparent K_m_ of HK for glucose 0.195 ± 0.012 mM was in a similar range as previously reported apparent K_m_ values ranging from 0.100 to 0.235 ± 0.041 mM, while the apparent K_m_ values of HK for ATP varied more strongly and ranged between 0.063 ± 0.004 and 2.857 ± 0.098 mM with our own measurement being 1.107 ± 0.074 mM (Viola et al., 1982; Golbik et al., 2001; Gao and Leary, 2003).

Several ATP regeneration systems have been described in literature including several polyphosphate kinase activities (Andexer and Richter, 2015; Tavanti et al., 2021). Among the enzymes that were successfully used, was the thermostable PPK from *Thermotoga maritima* (Honda et al., 2010; Kim and Zhang, 2016). One of the advantages of the thermostable enzyme is that for production host enzymes can be thermally inactivated and denatured, while the thermostable enzymes remain intact. We utilized the PPK2 enzyme for ATP regeneration that was successfully used by Rexer et al. (2018). Our study showed an activation of PPK2 activity by ADP and an inhibition by polyphosphate at concentrations higher than 10 mM while retaining 44% of the maximal activity at 50 mM polyphosphate. The inhibitory effect of polyphosphate in is in line with earlier reports, where inhibition was observed at concentrations higher than 6 mM (Rexer et al., 2018), however due to different qualities and properties of polyphosphate used, linear short chain polyphosphate versus metaphosphates, the values need to be considered with caution. Moreover, the observed inhibition of PPK2 is also indicative that the ATP regeneration system cannot be scaled up indefinitely if operated in a batch mode. The polyphosphate used in this study was Graham’s salt, which is a mixture of sodium metaphosphates. To our knowledge this is the first report showing that *P*. *aeruginosa* PPK2 can accept metaphosphate as a substrate, however, earlier research showed that *E*. *coli* PPK1 can accept metahexaphosphates as a phosphoryl donor (Murata et al., 1988).

As an alternative for the HK/PPK2 system for glucose 6-phosphate production, other enzyme activities could be considered. The polyphosphate-dependent glucokinase from *Thermobifida fusca* converted glucose into glucose 6-phosphate using linear polyphosphates as the phosphate donor (Liao et al., 2012). While the native enzyme was found to be rather unstable, an enzyme with enhanced thermostability and activity was generated (Zhou et al., 2018) and the enzyme was successfully applied in an enzyme cascade leading to formation of myo-inositol (Meng et al., 2018).

Finally, in a proof-of-concept study, we expanded the two-enzyme system by incorporating an enzymatic cellulose hydrolysis step that supplied glucose (Figure 5B). This set-up demonstrated the potential for valorization of a renewable feedstock into the value-added product glucose 6-phosphate. During the initial phase of the work to integrate the cellulose hydrolysis into the process, we explored the use of two model substrates, microcrystalline cellulose and Whatman paper, respectively. Both substrates enabled production of glucose 6-phosphate and thus this finding supports a certain flexibility of the three-enzyme system in terms of substrates to be accepted. However, due to a lower efficiency the use of Whatman paper as an alternative model substrate was stopped. We attributed this reduced efficiency to the fact that during the hydrolysis reaction a very viscous solution was produced. Compared to the two-enzyme system the glucose 6-phosphate yield from the cellulosic feedstock was quite modest. We tested different cellulose and cellulase concentrations to adjust the glucose release rate to match the HK/PPK2 glucose 6-phosphate production rate, however, we did not reach a higher conversion rate, though higher glucose 6-phosphate concentrations were obtained (data not shown). It is noteworthy that in addition to glucose and other hexoses, yeast hexokinases was shown to phosphorylate also cellulose (Purich et al., 1973; Božič et al., 2014). Thus, the presence of cellulose in the reaction might also lead to the phosphorylation of cellulose.

While our experiments indicated the possibility of a fully enzymatic one-pot production of glucose 6-phosphate, a chemo-enzymatic two stage process, in which acid hydrolysis is used to breakdown cellulose into cellooligosaccharides, followed by the enzymatic breakdown and conversion into the product could be considered as an alternative as exemplified by Meng et al. (2018).

**FIGURE 6:**
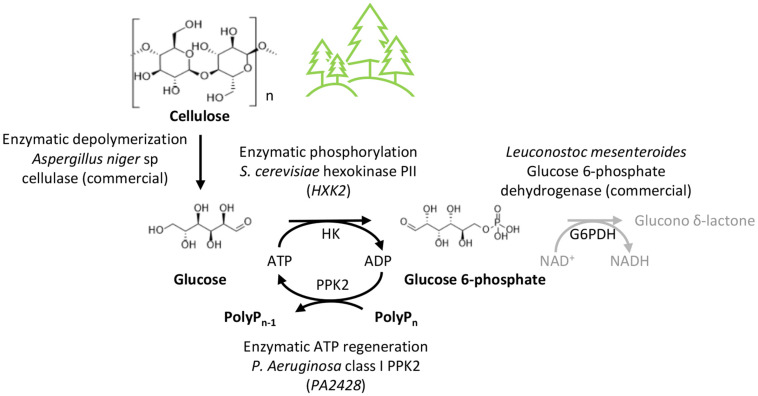
Conversion of cellulose into value-added products. Overall reaction scheme for the one-pot production of glucose 6-phosphate from cellulose. The glucose 6-phosphate dehydrogenase (G6PDH) reaction could be integrated into the reaction scheme for conversion into 6-phospho-D-glucono-1,5-lactone. Various other metabolites can be derived from glucose 6-phosphate.

Besides potentially accepting different substrates, the three-enzyme reaction can also be extended by a fourth reaction (Figure 6). In order to determine the reaction velocity of the three-enzyme reaction, we coupled the two-enzyme reaction to the interconversion of glucose 6-phosphate into 6-phospho-D-glucono-1,5-lactone by glucose 6-phospate dehydrogenase that enabled the kinetic study of the glucose 6-phosphate production by following the NADH formation of the coupled reaction. This demonstrates that a fourth reaction could be incorporated into this reaction scheme. As the reaction catalyzed by glucose 6-phospate dehydrogenase consumes NAD^+^, incorporation of this additional step would require an efficient method to regenerate NAD^+^ from NADH for which enzymatic or electrochemical reactions could be utilized (Wang X. et al., 2017).

## Conclusion

Cellulosic feedstocks are widely available and enable through either enzymatic routes or fermentation the production of a wide number of value-added biochemicals. Here, we explored the potential for enzymatic conversion of glucose released from cellulose into the model product glucose 6-phosphate using ATP as phosphoryl donor. Enzymatic conversions requiring cofactors are demanding due to the high costs and availability of cofactors. We successfully coupled the enzymatic phosphorylation reaction to a cofactor regeneration system. Using this system, a maximal conversion rate of 77% was obtained in the one pot synthesis of glucose 6-phosphate.

## Data Availability Statement

The original contributions presented in the study are included in the article/supplementary material, further inquiries can be directed to the corresponding author/s.

## Author Contributions

AF and AU conceived the study. AU and HL cloned, produced, and characterized the enzymes used in this study. HL characterized the thermostability of the enzymes using DSF, optimized the conditions for the final one-pot reaction. AU set-up the HPAEC analysis and established the initial conditions for the one-pot reactions. AU, HL, and AF analyzed the data. AF wrote the manuscript with help of AU and HL. All authors approved the final version of the manuscript.

## Conflict of Interest

The authors declare that the research was conducted in the absence of any commercial or financial relationships that could be construed as a potential conflict of interest.

## Footnotes

1 https://pubchem.ncbi.nlm.nih.gov/patent/GB-593480-A
